# Mixture Compound Fertilizer and Super Absorbent Polymer Application Significantly Promoted Growth and Increased Nutrient Levels in *Pinus massoniana* Seedlings and Soil in Seriously Eroded Degradation Region of Southern China

**DOI:** 10.3389/fpls.2021.763175

**Published:** 2021-12-07

**Authors:** Lanhua Mao, Ruibo Zha, Shifa Chen, Jing Zhang, Ling Jie, Xuan Zha

**Affiliations:** ^1^School of Geographical Sciences, Fujian Normal University, Fuzhou, China; ^2^Key Laboratory for Subtropical Mountain Ecology (Ministry of Science and Technology and Fujian Province Funded), Fujian Normal University, Fuzhou, China; ^3^College of Tourism, Fujian Normal University, Fuzhou, China; ^4^College of Tourism & Geography, Shaoguan University, Shaoguan, China; ^5^Institute of Geography, Fujian Normal University, Fuzhou, China

**Keywords:** *Pinus massoniana*, fertilisation, super absorbent polymer, nutrient uptake efficiency, N:P ratio

## Abstract

*Pinus massoniana* is the pioneer tree species in the red soil regions of southern China, however, the serious understory soil erosion and nutrient deficiency in that region are the main factors restricting the growth of *P. massoniana.* This field study examined the effects of compound fertilizer and super absorbent polymer (SAP) on the physiology, growth characteristics, biomass, soil nutrient, plant nutrient content, and nutrient uptake efficiency of 1-year-old *P. massoniana* seedlings for 2 years at Changting, Fujian in South China. One control (no fertilizer, CK) and fertilization treatments were established, namely, single compound fertilizer application (0.94, 1.89, and 3.56 g⋅plant^–1^) and mixture compound fertilizer and SAP application (0.94 + 1.01, 1.89 + 1.01, and 3.56 + 1.01 g⋅plant^–1^). Fertilization significantly improved the physiological performance, root collar diameter growth, height growth, biomass, and nutrient uptake of the seedlings. Compared with other fertilization treatments, the mixture compound fertilizer and SAP application significantly improved the seedling photosynthesis, which meant that the SAP had a significant effect on promoting photosynthesis. Under the mixture compound fertilizer and SAP application, the whole biomass of the seedlings was higher than that of all other treatments. Fertilization significantly increased the nitrogen (N), phosphorus (P), and potassium (K) content in the soils, leaves, stems, and roots of the seedlings, respectively. The P content was the main factor affecting growth characteristics and contributed to 58.03% of the total variation in seedling growth characteristics (*P* < 0.01). The N:P ratio of CK in the soils, leaves, and stems were higher than that of all the fertilization treatments, indicating that the severely eroded and degraded region had little P and required much of P. The principal component analysis indicated that the F2S (1.89 + 1.01 g) was the optimum fertilization amount and method in this experiment. These results provide a theoretical basis for the fertilization management of *P. massoniana* forests with severely eroded and degraded red soil regions.

## Introduction

As one of the severe global environmental problems, soil erosion hinders the sustainable development of the economy, society, and environment (Quinton et al., 2010). Soil erosion reduces the soil nutrient content, alters the soil structure, decreases the effective rooting depth of vegetation, and has a negative effect on vegetation growth (Jiang et al., 2020).

The southern red soil regions in China experience the most severe and widespread soil erosions in the country, with an erosion intensity and scale only second to those in the Loess Plateau (Chen Y. S. et al., 2020). Changting County in Fujian Province is a typical representative of severely eroded red soil in southern China (Zhu, 2013; Chen et al., 2019). At present, the area of soil erosion in the whole county measures 322.49 km^2^. It is well known that vegetation restoration is the main way to restore degraded ecosystems (Smith et al., 2014). *Pinus massoniana* is a pioneer tree species in the severely eroded and degraded land in the red soil region of South China, with developed roots, barren resistance, drought resistance, and slight acid preference, which plays an important role in forestry production and plantation ecosystem of China (Song et al., 2017; Qiao et al., 2019; Chen X. Z. et al., 2020). More than 5.7 × 10^7^ ha of *P. massoniana* plantations have been estimated in the southern part of China (Zhang et al., 2013). Previous studies have shown that the canopy density of *P. massoniana* forests can increase canopy interception (Chen et al., 2011), reduce soil erosion, and enhance the soil and water conservation ability (Cao et al., 2008; Zhao et al., 2019; Sui et al., 2021). In addition, *P. massoniana*, as a fast-growing and high-yield timber tree species, plays an important role in increasing the total amount of forest resources (Sui et al., 2021). However, artificial damage and poor soil fertility restrict the growth of *P. massoniana*, resulting in a decrease in productivity. Therefore, to improve the fast-growing and high-yield of *P. massoniana*, fertilization is one of the effective treatments (Zeng et al., 2013).

In China, active forest fertilization was initiated as early as the 1950s. However, there is limited literature on the fertilization of *P. massoniana*. Moreover, previous studies have shown that fertilization could evidently promote the growth and nutrient uptake efficiency of *P. massoniana* as well as the physicochemical properties of the soil (Zheng et al., 2015; Zhang et al., 2018; Jiang et al., 2019). For instance, soil and foliar nitrogen (N) application promoted the growth of *P. massoniana* to varying degrees (Huang et al., 2019), while the supplementation of moderate-to-high N significantly increased soil and foliar N content (Huang et al., 2019; Wu et al., 2019). The photosynthetic rate of plants is closely related to N; therefore, the increase in foliar N content with soil N fertilization increased the photosynthetic rate of *P. massoniana* (Harrison et al., 2010). In a study of *P. massoniana* in a subtropical forest, Yang (2018) found that phosphorus (P) is limited in subtropical soils; therefore, soil P supplementation promoted *P. massoniana* growth. Similarly, through a 3-year fertilization experiment on *P. massoniana*, Xiao and Lan (1998) found that P fertilization was the best treatment, which increased plant height by 20.5–22.2% and stem diameter by 19.8–20.8%. Finally, Zhao et al. (2016) and Wang et al. (2017) noted that phosphate fertilization substantially improved the growth of *P. massoniana* seedlings.

In general, the main factor affecting the plants growth and their quality are quantity of water and fertilizers that can be absorb by plants (Liu et al., 2013; Ghazali et al., 2016). In red soil regions, acid soils can lead to the lack of numerous essential plant nutrients, especially low phosphorus and nitrogen availability are the limiting factor of plant growth (Zhang et al., 2013; Chen et al., 2018). Moreover, Phosphorus and nitrogen can affect various important metabolic processes in plants, such as energy transport, photosynthesis and respiration (Khan et al., 2014; Chen et al., 2018), and red soils in Fujian Province are developed from granite, the phosphorus and nitrogen content of which is low (Zeng et al., 2013). On the other hand, soils in the area are mostly characterized by low water-holding capacity, high evapo-transpiration and excessive leaching of the rainfall, leading to poor water and fertilizer use efficiency by plants (Islam et al., 2011). Super absorbent polymer (SAP) as a novel approach has a very high water absorption and retention capacity, absorbing the amount of water hundreds or even thousands of times its own mass, and can also absorb water repeatedly (Busscher et al., 2009; Suresh et al., 2018). The application of SAP for stabilizing soil structure resulted to increased infiltration, improved water use efficiency and reduced soil erosion (Fernando et al., 2017; Hou et al., 2018). When polymers are incorporated with soil, it is presumed that they retain large quantities of water and nutrients, which are released as required by the plant (Islam et al., 2011; Liu et al., 2013). The use of SAP as carrier and regulator of nutrient release was helpful in reducing undesired fertilizer losses, while sustaining vigorous plant growth (Islam et al., 2011).

Here, three types of fertilization treatments were applied to 1-year-old *P. massoniana* seedlings in the serious erosion region, namely, single compound fertilizer application and mixture compound fertilizer and SAP application, and their effects on the physiology, growth, biomass, nutrient allocation, and fertilizer uptake efficiency (FUE) of the seedlings were examined. The study aimed to (1) analyze the effects of different fertilization treatments on the photosynthesis, growth characteristics, and nutrient content in soils and plants; (2) calculate the nutrient uptake efficiency of seedlings based on nutrient content under different fertilization treatments and analyze their change trends; and (3) synthetically analyze the effects of fertilization on seedling indices using principal component analysis (PCA) and determine the optimal fertilization amount and method for *P. massoniana*. Our results will provide an effective theoretical basis for the fertilization management of *P. massoniana* growing in the eroded and degraded red soils of southern China.

## Materials and Methods

### Materials and Experimental Design

The present study was conducted at a *P. massoniana* forest in Hetian (25°18′40′′–26°02′05′′N, 116°00′45′′–116°39′20′′E) in Changting County, Fujian Province, China, representing a typical serious erosion soil region of southern China. The mean precipitation and annual temperature were 1,700 mm and 18.3°C, respectively. The geomorphology is mainly low mountains and hills. The soil type is granite red soil, having poor corrosion resistance and strong acidity. The stand is single, dominated by *P. massoniana.*

In this study, seven adjacent plots of 20 × 5 m^2^ were established in March 2018. The slope of plots was 15°. Each of the plots is surrounded by a partition of about 20 m. For each plot, 20 of the *P. massoniana* seedlings used in the experience were 1-year-old, with a root collar diameter of 1.84 ± 0.26 cm and a height of 16.74 ± 2.35 mm. The experiment was performed from March 2018 to September 2019. The seven plots were divided among seven treatments (Table 1), namely F1 (0.94 g compound fertilizer per plant), F2 (1.89 g compound fertilizer per plant), F3 (3.56 g compound fertilizer per plant), F1S (0.94 g compound fertilizer and 1.01 g SAP per plant), F2S (1.89 g compound fertilizer and 1.01 g SAP per plant), F3S (3.56 g compound fertilizer and 1.01 g SAP per plant), and CK (control, no fertilization), with one plot per treatment. Each treatment had 20 replications. Compound fertilizer was obtained from Quzhou Non-gdehui fertilizer Technology Co., Ltd. (N:P:K = 16:5:10). Super absorbent polymer (Polyacrylamide, small white particles measuring 0.5 mm; water absorption rate = 275.88) was purchased from Beijing Hanlimiao Co. Before the treatments, there was no difference in the soil chemistry. Except for the fertilization factor, all other conditions were the same in this experiment, watering once a week.

**TABLE 1:** Experimental treatments.

Treatments	Compound fertilizer	SAP
CK	0	0
F1	0.94	0
F2	1.89	0
F3	3.56	0
F1S	0.94	1.01
F2S	1.89	1.01
F3S	3.56	1.01

### Measurements

The plant height growth (HG) and root collar diameter (RCD) were regularly measured every month on March 22, 2018. The HG was measured using a steel tape gauge with an accuracy of.1 cm, and the RCD was measured using a Vernier caliper with an accuracy of.01 mm.

The photosynthetic rate of the seedlings was measured on July 18, 2018 and July 18, 2019. To reduce the influence of light conditions and select sunny days, the LI-6800 portable photosynthetic meter was used, and measurements were obtained from 9:00 to 11:00. The intensity of photosynthetic radiation was set at 2,000 mol⋅m^–2^⋅s^–1^. The net photosynthetic rate (*P*_*n*_), transpiration rate (*T*_*r*_), and stomatal conductance (*G*_*s*_) of the *P. massoniana* seedlings were measured. The water use efficiency (*WUE*) was calculated using the following equation (Li et al., 2017; Zhou et al., 2020):


(1) WUE=PTn/r

On September 15, 2019, the soil samples were collected from the 0–10, 10–20, and 20–40 cm soil layers at each site. For each treatment plot, 10 soil samples were collected. Air-dried soil samples were used to determine the soil physicochemical properties. The 10 seedling samples were selected from the treatments for destruction sampling on September 15, 2019. The entire seedling was taken out of the pot and placed in water, gently shaking the soil to separate it from the roots. Thereafter, the roots were washed with deionized water. The roots, stems, and leaves of the whole seedlings were separated; placed in envelope bags; and dried in an oven at 105°C for 1 h and then at 65°C to a constant weight, followed by dry weight measurement. The dried samples were then ground for the analysis of seedling nutrient content.

Soil total nitrogen (TN) content was determined using the vario Max Element Analyzer (Germany) and plant nitrogen (N) was using the vario EL III Element Analyzer (Germany), respectively. Following digestion with H_2_SO_4_–HClO_4_, soil total phosphorus (TP) and plant phosphorus (P) content were determined using the Skalar SAN + ⁣ + continuous flow analyzer (Netherlands). The availability of soil nutrients (AP and AK) are fundamental indexes needed to evaluate soil quality. Soil available phosphorus (AP) was extracted with 0.5 mol⋅L^–1^ NaHCO^3^, then determined by molybdenum-antimony colorimetry. Soil total potassium (TK) and available potassium (AK) content were determined by the FP640 flame photometer (Shanghai Xin Yi Precision Instrument Co., LTD). The FUE for the N, P, and potassium (K) content of the seedlings was calculated using the following equation (Erro et al., 2011; Zeng et al., 2013; Karami et al., 2020):


(2) FUE(%)=(N-FN)U/FN×100

where N_*F*_ is the nutrient (N, P, or K) content of the seedlings with fertilizer application; N_*U*_ is the nutrient (N, P, or K) content of the seedlings without fertiliser application (CK); and FN is the fertilizer (N, P, or K) applied in the treatment.

### Statistical Analysis

The significance of differences among the photosynthesis, growth characteristics, biomass, nutrient content, and nutrient use efficiency of the seedlings was analyzed by one-way ANOVA and then the averages were compared by Tukey’s test in the IBM SPSS Statistics 19.0 software (Armonk, NY, United States). The physiological and growth characteristics, biomass, and nutrient content of the seedlings under different treatments were analyzed by PCA using the Origin 2018 software (Origin Lab Inc., Northampton, MA, United States). To comprehensively evaluate the effects of different treatments on the seedlings, PCA was performed in IBM SPSS Statistics 19.0 software. The redundancy analysis (RDA) was using the CANOCO 5.0 software (Ter Braak and Smilauer, 2002) for exploring the relationships between the growth characteristics and nutrient content variables tested.

## Results

### Rainfall Characteristics During the Experimental Period

**FIGURE 1:**
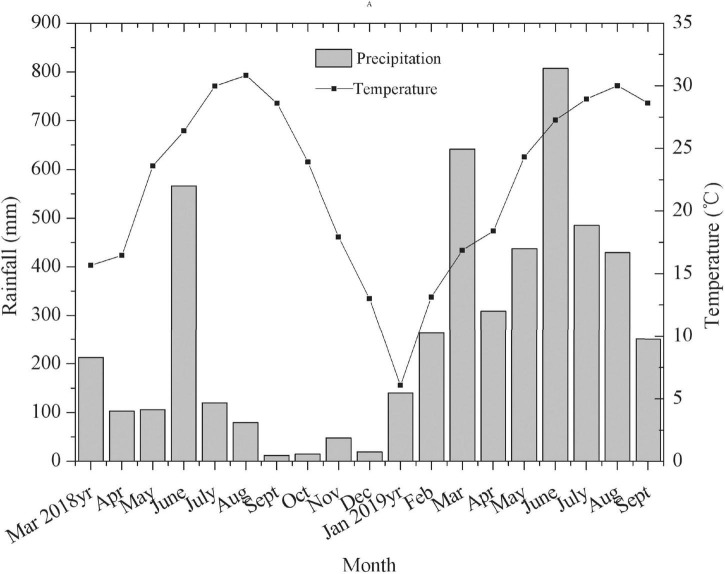
The distribution diagram of the monthly mean rainfall and temperature in the runoff plot from March 2018 to August 2019.

During experimental period, the monthly mean rainfall and temperature data from March 2018 to September 2019 were obtained from RG3-M rain gauge (United States) in runoff plot (Figure 1). The distribution of monthly mean rainfall and temperature were uneven, with the highest rainfall in June and highest temperature in August (Figure 1).

### Physiological Characteristics

Fertilization significantly increased the *P*_*n*_, *G*_*s*_, *T*_*r*_, and WUE of the *P. massoniana* seedling needles compared with the control (*P* < 0.05) (Table 2). However, *P*_*n*_ did not increase with increasing fertilizer application. As such, under the single compound fertilizer application, the order of *P*_*n*_ was F2 > F3 > F1 in July 2018 and F2 > F1 > F3 in July 2019. The *P*_*n*_ under F2S was significantly higher than that of the remaining treatments. However, under the same treatment, the *P*_*n*_ of the seedlings varied greatly across different periods. The highest *P*_*n*_ was recorded in July 2018, followed by July 2019 (Table 2).

**TABLE 2:** Net photosynthetic rate, stomatal conductance, transpiration rate, and water use efficiency of *Pinus massoniana*seedling needles under different treatments.

Treatments	Net photosynthetic		Stomatal conductance		Transpiration rate		Water use efficiency	
	(μmol m^–2^ s^–1^)		(mol m^–2^ s^–1^)		(mmol m^–2^ s^–1^)		(μmol m^–2^ s^–1^)	
	
	2018–2007	2019–2007	2018–2007	2019–2007	2018–2007	2019–2007	2018–2007	2019–2007
F1	7.12 ± 0.19^d^	6.19 ± 0.30^c^	0.18 ± 0.00b^c^	0.11 ± 0.00^c^	0.0034 ± 0.0001^c^	0.0014 ± 0.0001^e^	2.13 ± 0.03^c^	4.52 ± 0.02^b^
F2	8.52 ± 0.16a^b^	7.50 ± 0.19^b^	0.19 ± 0.00^b^	0.13 ± 0.00^b^	0.0037 ± 0.0001^ab^	0.0016 ± 0.0001^b^	2.30 ± 0.03^b^	4.66 ± 0.10^b^
F3	8.24 ± 0.00b^c^	5.76 ± 0.14^c^	0.17 ± 0.00^c^	0.11 ± 0.00^c^	0.0036 ± 0.0001^b^	0.0014 ± 0.0001^e^	2.28 ± 0.05^b^	4.24 ± 0.03^c^
F1S	7.94 ± 0.10^c^	6.19 ± 0.20^c^	0.16 ± 0.00^c^	0.11 ± 0.00^c^	0.0033 ± 0.0001^c^	0.0015 ± 0.0001^d^	2.4 ± 0.10^a^	4.27 ± 0.11^c^
F2S	9.15 ± 0.16^a^	8.28 ± 0.27^a^	0.21 ± 0.00^a^	0.14 ± 0.01^a^	0.0038 ± 0.0001^a^	0.0017 ± 0.0001^a^	2.41 ± 0.14^a^	4.84 ± 0.30^a^
F3S	8.54 ± 0.19^ab^	7.11 ± 0.14^b^	0.17 ± 0.00b^c^	0.12 ± 0.00^b^	0.0037 ± 0.0001^b^	0.0016 ± 0.0001^bc^	2.34 ± 0.09^ab^	4.53 ± 0.05^b^
CK	5.05 ± 0.07^e^	3.72 ± 0.12^d^	0.08 ± 0.01A^e^	0.07 ± 0.00^d^	0.0026 ± 0.0001^e^	0.0011 ± 0.0001^f^	1.98 ± 0.10^d^	3.38 ± 0.11^d^

*Values are presented as mean ± standard error from 10 replicates each treatment. Different letters indicate significant differences among treatments (Tukey’s test, P ≤ 0.05).*

Similar to the *P*_*n*_, the *G*_*s*_ and *T*_*r*_ of the seedlings did not increase with the increasing fertilizer application. The *G*_*s*_ and *T*_*r*_ under F2S were the highest, and there were significant differences in values between F2S and the remaining treatments (*P* < 0.05). Under the same treatment, the *G*_*s*_ and *T*_*r*_ of the seedlings were the highest in July 2018, being significantly higher than the values in July 2019 (Table 2).

Contrary to the *P*_*n*_, *G*_*s*_, and *T*_*r*_, the highest WUE of the seedlings was recorded in July 2019 under the same treatment, and this value was significantly higher than that in July 2018. The WUE was the lowest in July 2018, indicating that the WUE decreased as the *P*_*n*_, *G*_*s*_, and *T*_*r*_ increased (Table 2).

### Growth Characteristics and Biomass Allocation

**FIGURE 2:**
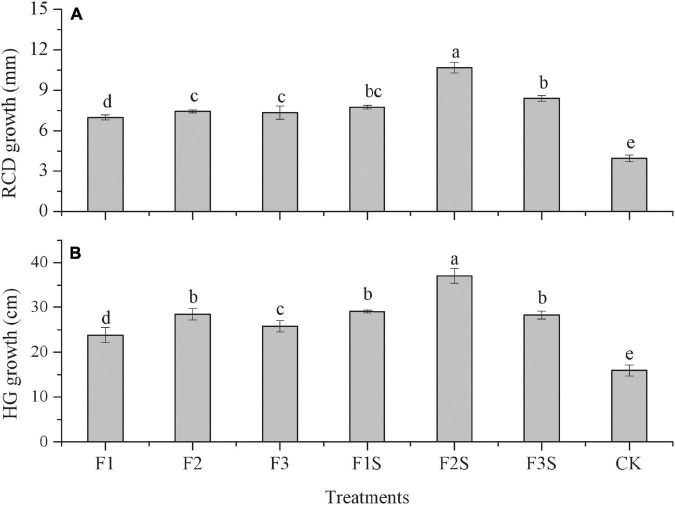
The root collar diameter (RCD) growth **(A)** and height growth (HG) **(B)** of *P. massoniana* seedlings under different treatments. Values are presented as mean ± SE from 10 replicates per treatment. Different letters indicate significant differences among treatments (Tukey’s test, *P* ≤ 0.05).

Fertilizer treatments significantly improved the RCD growth of the *P. massoniana* seedlings (*P* < 0.05) (Figure 2A). The RCD growth of the seedlings under different fertilizer treatments ranged from 5.56 to 10.68 mm and was significantly higher than that under CK (3.95 mm) (*P* < 0.05). The one-way ANOVA indicated significant differences between the fertilization and control treatments (*P* < 0.05). Among the different fertilization treatments, the maximum RCD growth of the seedlings was obtained under F2S, and the value under this treatment was significantly higher than that under the remaining treatments (*P* < 0.05) (Figure 2A). The RCD growth under F1 and F3 was significantly lower than that under F2 (Figure 2A).

Moreover, fertilization significantly affected the HG of the seedlings (*P* < 0.05). The HG of the seedlings under different fertilization treatments ranged from 22.32 to 37.07 cm, which was significantly higher than that under CK (15.90 cm). The HG under F2S was significantly higher than that under the remaining treatments (Figure 2B). The HG under F2 was significantly higher than that under F1 and F3 (Figure 2B).

**FIGURE 3:**
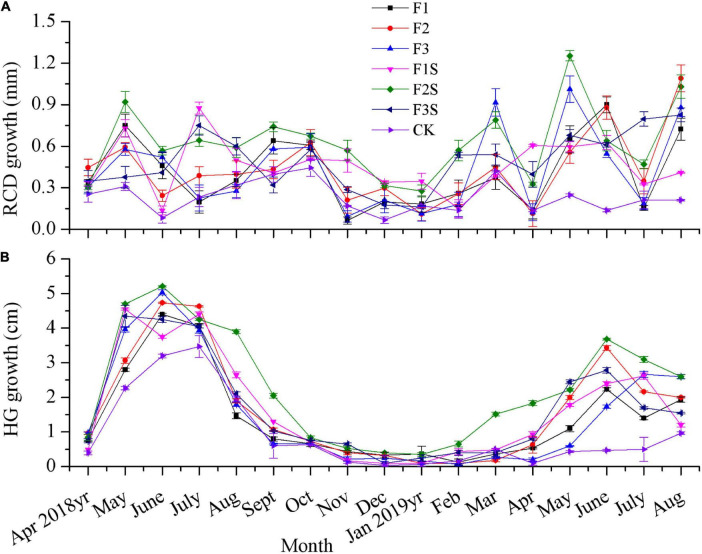
Monthly increased growth in the RCD **(A)** and HG **(B)** of *P. massoniana* seedlings under different treatments.

The monthly increased growth of the seedlings in terms of RCD and HG differed across the fertilizer treatments (Figure 3). Before fertilization, the RCD growth and HG of the seedlings were relatively slow. One month after fertilization, the RCD growth and HG of the seedlings increased rapidly (Figure 3). The RCD growth of the seedlings increased at a uniform rate from June 2018 to November 2018 and slowed, or even stopped, from December 2018 to February 2019. In March 2019, the RCD growth became rapid again, reaching a peak in May. The highest RCD growth was achieved under F2S (1.25 mm), and growth under this treatment was five times that under CK (Figure 3A). The HG of the seedlings was rapid from June 2018 to September 2018 and slowed, or even stopped, from October 2018 to February 2019. After March 2019, the HG began to increase rapidly, reaching a peak in June, although the growth rate was not very high (Figure 3B). The maximum HG of the seedlings was achieved in June 2018 under F2S (5.21 cm), and the growth under this treatment was 2.72 times that under CK (Figure 3B). Overall, the monthly RCD growth and HG of the fertilized *P. massoniana* seedlings were higher than those of the non-fertilized ones (Figure 3).

Under different fertilizer treatments, the biomass of the roots, stems, and leaves of the seedlings increased significantly (Table 3). The root biomass of the fertilized seedlings (7.24–9.46 g) was significantly higher than that of CK (4.17 g). The highest root biomass was noted under F2S, and the value under this treatment was 2.27 times higher than the value under CK (Table 3). The root biomass under F2 and F3 was significantly higher than that under F1 (*P* < 0.05) (Table 3). The root biomass under F2 and F3 was significantly higher than that under F1 (*P* < 0.05). The root biomass under F2S and F3S was significantly higher than that under F1S (Table 3). However, the root biomass of the seedlings subjected to the single compound fertilizer application or mixture compound fertilizer and SAP application did not increase with increasing fertilization (Table 3).

**TABLE 3:** Dry biomass of roots, stems, leaves, and whole seedling biomass (g) in different treatments.

Treatments	Roots (g)	Stems (g)	Leaves (g)	Whole biomass (g)
F1	7.24 ± 0.16^d^	4.84 ± 0.18^d^	13.74 ± 1.16^d^	25.83 ± 1.29^e^
F2	8.98 ± 0.40^ab^	7.65 ± 0.16^c^	17.35 ± 0.58^b^	33.99 ± 1.57^c^
F3	8.77 ± 0.20^b^	7.74 ± 0.23^c^	14.96 ± 0.61^c^	31.48 ± 1.67^d^
F1S	7.87 ± 0.25^c^	8.54 ± 0.53^b^	17.14 ± 0.77^b^	33.55 ± 2.16^c^
F2S	9.46 ± 0.32^a^	9.71 ± 0.63^a^	20.35 ± 0.86^a^	39.52 ± 1.92^a^
F3S	8.80 ± 0.50^b^	9.83 ± 0.55^a^	17.49 ± 0.67^b^	36.11 ± 1.18^b^
CK	4.17 ± 0.13^e^	2.53 ± 0.24^e^	4.12 ± 0.25^e^	10.83 ± 0.51^f^

*Values are presented as mean ± standard error from 10 replicates each treatment. Different letters indicate significant differences among treatments (Tukey’s test, P ≤ 0.05).*

Contrary to the changing trend of the root biomass, the stem biomass of the *P. massoniana* seedlings increased with the increasing fertilization. The stem biomass under fertilization (4.53–9.83 g) was significantly higher than that under the control (*P* < 0.05) (Table 3). The highest stem biomass was achieved under F3S, and the value under this treatment was significantly higher than that under the remaining treatments, except that with F2S. The stem biomass under F2 and F3 was significantly higher than that under F1. The stem biomass under F2S and F3S was significantly higher than that under F1S (*P* < 0.05) (Table 3).

Furthermore, fertilization significantly affected the leaf biomass (*P* < 0.05). The leaf biomass of the fertilized seedlings was significantly higher than that of the CK (Table 3). However, the leaf biomass did not increase with the increasing fertilizations. The leaf biomass under the different fertilization treatments was in the order of F2S > F3S > F1S and F2 > F3 > F1. The leaf biomass under F2S was the largest, and the value under this treatment was significantly higher than that under the remaining fertilization treatments. The second largest leaf biomass was achieved under F3S, and the value under this treatment was significantly higher than that under the remaining fertilization treatments, except that in F1S and F2 (Table 3).

Under fertilization, the total biomass of the seedlings ranged from 25.83 to 39.52 g, which was twice the value under CK. The highest total biomass was achieved under F2S, and the value under this treatment was 3.58 times higher than that under CK and significantly higher than that under the remaining fertilization treatments (*P* < 0.05) (Table 3).

### The Changes in Soil Chemical Properties

**FIGURE 4:**
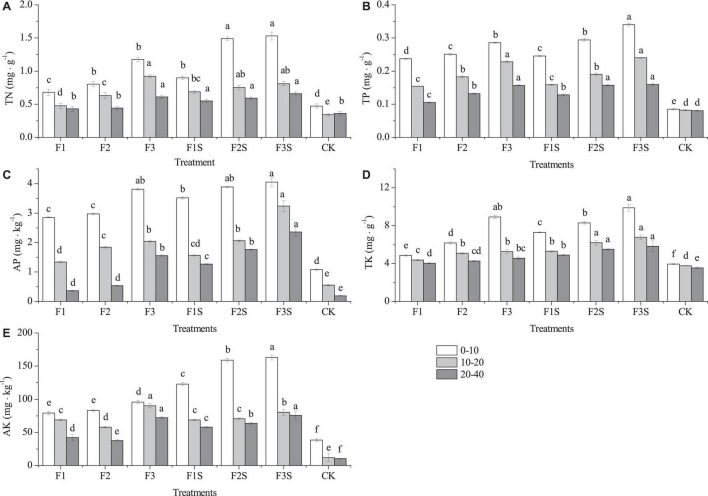
The content of total nitrogen (TN) **(A)**, total phosphorus (TP) **(B)**, available phosphorus (AP) **(C)**, total potassium (TK) **(D)**, and available potassium (AK) **(E)** characteristics in the soil for different fertilization treatments. Different letters indicate significant differences among treatments (Tukey’s test, *P* ≤ 0.05).

Compared to CK, fertilization significantly had significantly affected the content of TN, TP, AP, TK, and AK, and those of them increased with increasing fertilization (Figure 4). The TN, TP, AP, TK, and AK showed a gradually decreasing trend from top soil to deep soil layers (Figure 4). The TN, TP, AP, TK, and AK of F3S were higher than those of other treatments in 0–10 cm and 20–40 cm soil layers. F3S had highest TP, AP, and TK in 10–20 cm soil layer (Figures 4B–D), and F3 had highest TN and AK (Figures 4A,E).

### Nutrient Allocation

Fertilization significantly increased the N content of the leaves, stems, and roots of the seedlings, and these values were significantly higher than those under CK (*P* < 0.05); however, the N content in the leaves, stems, and roots did not increase with the increasing fertilization (Table 4). The highest N content in the leaves was achieved under F2S, and the value under this treatment was significantly higher than that under the remaining treatments (Table 4). The N content in leaves under F2 was significantly higher than that under the remaining treatments, except F2S and F3S. The N content under F1 in the leaves was the lowest, and the value under this treatment was significantly lower than that under the remaining treatments (*P* < 0.05) (Table 4). The N content in the stems and roots was the highest under F2S and the value under this treatment was significantly higher than that under the remaining treatments (*P* < 0.05), except F2 (*P* > 0.05) (Table 4). Under the same fertilization treatment, the N content in the leaves was significantly higher than that in the roots and stems, whereas the N content of the stems was significantly higher than that of the root (*P* < 0.05) (Table 4).

**TABLE 4:** N, P, and K content of *Pinus massoniana* seedlings under different fertilization treatments.

Nutrient allocation		Treatments							*p-*values
		F1	F2	F3	F1S	F2S	F3S	CK	
N(g kg^–1^)	Leaves	11.98 ± 0.38d	16.22 ± 0.53^b^	14.61 ± 0.64^c^	12.55 ± 0.48cd	18.45 ± 0.41^a^	15.83 ± 0.29^b^	9.50 ± 0.42^e^	<0.001
	Stems	5.14 ± 0.40^d^	8.35 ± 0.31^a^	6.77 ± 0.23^bc^	6.15 ± 0.33^c^	8.67 ± 0.55^a^	7.04 ± 0.11^b^	3.98 ± 0.22^e^	<0.001
	Roots	4.08 ± 0.24^b^	5.17 ± 0.28^a^	4.24 ± 0.23^b^	4.07 ± 0.30^b^	5.27 ± 0.26^a^	4.65 ± 0.21^b^	2.73 ± 0.16^c^	0.000
P(g kg^–1^)	Leaves	1.14 ± 0.06^e^	1.57 ± 0.03^bc^	1.44 ± 0.06^cd^	1.36 ± 0.05^d^	1.76 ± 0.04^a^	1.66 ± 0.04^ab^	0.93 ± 0.04^f^	<0.001
	Stems	0.51 ± 0.05^d^	0.74 ± 0.06^b^	0.69 ± 0.04^bc^	0.62 ± 0.03^c^	0.84 ± 0.05^a^	0.65 ± 0.04^c^	0.32 ± 0.05^e^	0.001
	Roots	0.33 ± 0.05^c^	0.41 ± 0.03^b^	0.37 ± 0.03^bc^	0.38 ± 0.03^b^	0.51 ± 0.01^a^	0.43 ± 0.03^b^	0.26 ± 0.03^d^	<0.001
K(g kg^–1^)	Leaves	3.60 ± 0.22^c^	4.81 ± 0.12^b^	4.68 ± 0.08^b^	4.05 ± 0.06^bc^	6.02 ± 0.09^a^	4.94 ± 0.04^b^	2.93 ± 0.16^d^	<0.001
	Stems	3.3 ± 0.43^c^	4.59 ± 0.29^b^	4.25 ± 0.21^b^	4.26 ± 0.15^b^	5.27 ± 0.30^a^	4.71 ± 0.19^ab^	2.35 ± 0.20^d^	<0.001
	Roots	2.79 ± 0.14^e^	3.78 ± 0.29^d^	3.55 ± 0.26^d^	4.02 ± 0.08^cd^	5.19 ± 0.19^a^	4.53 ± 0.25^bc^	2.29 ± 0.15^e^	<0.001

*Values are presented as mean ± standard error from 10 replicates each treatment. Different letters indicate significant differences among treatments (Tukey’s test, P ≤ 0.05).*

Under the same fertilization treatment, the P content of the seedlings was in the order of leaf > stem > root (Table 4). Among the different fertilization treatments, the highest leaf P content was achieved under F2S, and the value under this treatment was significantly higher than that under the remaining treatments (*P* < 0.05), except for that in F3S. The P content of the leaves under F1 was significantly lower than that under F2 and F3 (Table 4). The stem P content under F2S was significantly higher than that under the remaining treatments, except for that under F2 and F3S (*P* < 0.05) (Table 4). The root P content under F2S was significantly higher than that under the remaining treatments (*P* < 0.05). The root P content under F1 was significantly lower than that under F2 and F3 (Table 4).

Under the same fertilization treatment, the K content of the seedlings was in the order of root > leaf > stem (Table 4). The K content of the leaves, stems, and roots was the highest under F2S. The leaf K content under F2S was significantly higher than that under the remaining treatments (*P* < 0.05). The leaf K content under F1 was significantly lower than that under F2 and F3. The stem K content under F2 was significantly higher than that under F1 and F3. The stem K content under F2S was significantly higher than that under F1S, although there was no significant difference in the stem K content between F2S and F3S (*P* > 0.05) (Table 4). The root K content under F2S was significantly higher than that under the remaining treatments (Table 4). The root K content under F1 was significantly lower than that under F2 and F3. Overall, the N, P, and K content of the P. massoniana seedlings were in the order of leaf > stem > root, and the nutrient content of the leaves, stems, and roots was in the order of N > K > P (Table 4).

### The N:P Ratio of Soil, Leaves, Stems, and Roots

**FIGURE 5:**
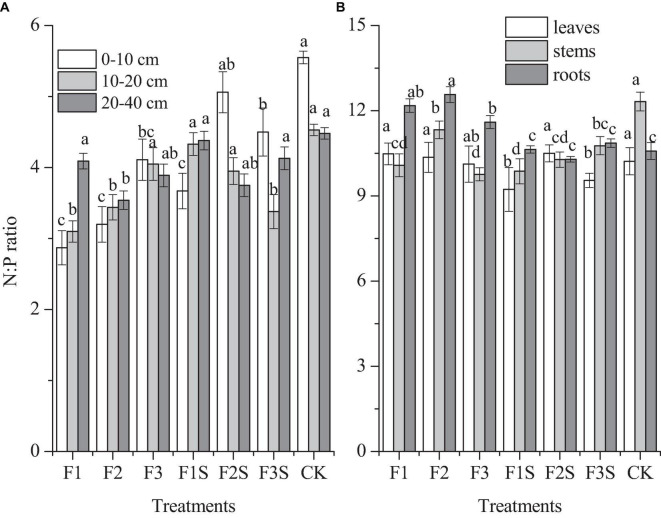
The N:P ratio of soil **(A)**, leaves, stems, and roots **(B)** for different treatments. Different letters indicate significant differences among treatments (Tukey’s test, *P* ≤ 0.05).

The N:P ratios in 0–10, 10–20, and 20–40 cm soil layers were 2.87–5.55, 3.10–4.33, 3.34–4.48, respectively (Figure 5A). Compared to fertilization treatments, CK had highest N:P ratio (Figure 5A). In general, N:P ratio in soils was increased with increasing fertilizer addition, and increased with the depth of soil layer under single compound fertilizer application (Figure 5A). F2S had significantly higher N:P ratio than all other fertilizer treatments in 0–10 cm soil layer (Figure 5A).

The N:P ratios in leaves, stems, and roots were 9.23–10.50, 9.76–12.32, 10.29–12.57, respectively (Figure 5B). All F2S had higher N:P ratio in leaves and had lower N:P ratio in roots than that all other treatments (Figure 5B). The N:P ratio of F2 was higher than that of other treatments in roots, and that of CK was was higher than that of other treatments in stems (Figure 5B).

### Fertilizer Uptake Efficiency

Fertilization significantly affected the N uptake efficiency (NUE) of the seedlings (*P* = 0.000) (Table 5). NUE of the seedlings varied under different fertilization treatments, although it did not increase with increasing fertilization. The NUE under F2 was 1.35 times the value under F1 and 2.71 times the value under F3. NUE under F2S was the highest, being 1.22 times the value under F1S and 2.70 times the value under F3S (Table 5).

**TABLE 5:** Nutrient uptake efficiency of *Pinus massoniana* seedlings under different fertilization treatments.

Treatments	Nutrient absorption efficiency (%)		
	*N*	*P*	*K*
F1	33.13 ± 3.81^c^	10.17 ± 1.75^c^	19.38 ± 1.47^c^
F2	44.74 ± 0.47^b^	12.75 ± 1.23^b^	28.11 ± 3.56^b^
F3	16.52 ± 1.35^d^	5.57 ± 0.60^e^	12.95 ± 1.28^d^
F1S	43.64 ± 4.83^b^	16.96 ± 1.63^a^	45.57 ± 2.47^a^
F2S	53.54 ± 3.36^a^	18.22 ± 0.89^a^	47.40 ± 1.97^a^
F3S	19.84 ± 0.38^d^	6.90 ± 0.59^d^	17.75 ± 1.35^c^
*p* values	0.000	0.000	0.000

*Values are presented as mean ± standard error from 10 replicates each treatment. Different letters indicate significant differences among treatments (Tukey’s test, P ≤ 0.05).*

The P uptake efficiency (PUE) of the seedlings was also affected by fertilization (*P* = 0.000) (Table 5). PUE under F1 was 83% greater than that under F3. PUE under F2 was 129% greater than that under F3. In addition, the highest PUE was achieved under F2S, and the value under this treatment was significantly higher than that under the remaining fertilization treatments, except F1S (Table 5).

Likewise, fertilization significantly affected the K uptake efficiency (KUE) of the seedlings (*P* = 0.000) (Table 2). KUE under F2 was 1.45 and 2.17 times that under F1 and F3, respectively. The highest KUE was achieved under F2S, and the value under this treatment was significantly higher than that under the remaining treatments, except F1S (Table 5).

### Comprehensive Analysis

**FIGURE 6:**
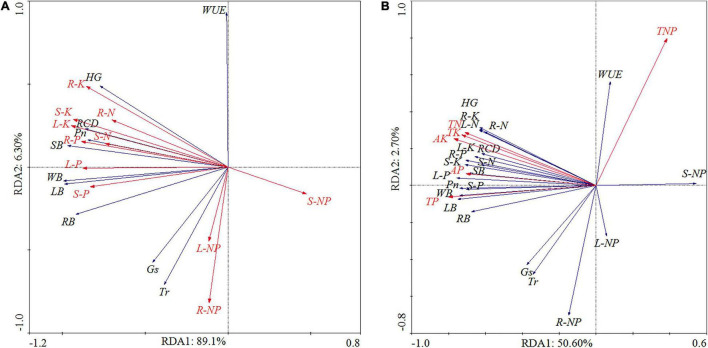
Principal components analysis of all parameters under different fertilization treatments. Note: The relationship between the photosynthesis, growth characteristics, and nutrient content of the *P. massoniana* seedlings **(A)**; The relationship between the soil nutrient and the photosynthesis, growth characteristics, and nutrient content of the *P. massoniana* seedlings **(B)**. Net photosynthetic rate (*P*_*n*_), stomatal conductance (*G*_*s*_), transpiration rate (*T*_*r*_), water use efficiency (WUE), height growth (HG), root collar diameter growth (RCD), root biomass (RB), stem biomass (SB), leaves biomass (LB), whole biomass (WB), nitrogen (N), phosphorus (P), and potassium (K).

The relationship between the photosynthesis, growth characteristics, and nutrient content of the *P. massoniana* seedlings were examined by redundancy analysis (RDA) (Figure 6A). The results showed that all considered nutrient content variables of *P. massoniana* seedlings significantly explained 95.40% of the total variation in photosynthesis and growth characteristics (Figure 6A). The N, P, and K in the leaves, stems, and roots were responsible for a lot of the variations in growth characteristics. The RDA showed that the TN, TP, AP, TK, and AK in the soil had a significantly positive correlation with the *P*_*n*_, RCD, HG, LB, SB, RB, WB, and nutrient content in the seedlings (Figure 6B). In particular, the P in the leaves was the first factor that contributed to 58.03% of total variation (*P* < 0.01), and the TP and AP in the soil were the vital factor that contributed to more 38.26% of the total variation (Figure 6A,B). In addition, the N:P ratio in the soil was significantly negatively correlated with the *P*_*n*_, *G*_*s*_, *T*_*r*_, RCD, HG, LB, SB, RB, WB, and nutrient content in the seedlings, but had a significantly positive correlation with WUE (Figure 6B).

According to the PCA of parameters under the different fertilization treatments, the highest coefficient for PC1 was recorded under the F2S treatment, and the value under this treatment was significantly higher than that under the remaining treatments (Table 6). The coefficient for PC1 under F3S and F2 was significantly higher than that under CK, F1, F3, and F1S. The coefficient for PC1 under fertilization was significantly higher than that under CK (Table 6). The highest coefficient for PC2 was recorded under F2S, and the value under this treatment was significantly higher than that under the remaining treatments, except F1S (Table 6). The results of the comprehensive analysis under the different fertilization treatments revealed that the comprehensive score for F2S was the highest, followed by that for F3S, indicating that the mixture compound fertilizer and SAP application is highly suitable for *P. massoniana* seedling growth (Table 6).

**TABLE 6:** Scores and ranks of principal components and comprehensive scores for fertilizer treatments.

Treatments	PC1		PC2		Comprehensive	
	Score	Rank	Score	Rank	Score	Rank
CK	−6.61 ± 0.21^g^	7	0.95 ± 0.16^bc^	3	−4.76 ± 0.19^g^	7
F1	−1.18 ± 0.46^ef^	6	−2.39 ± 0.43^e^	7	−1.48 ± 0.25^f^	6
F2	2.05 ± 0.02^b^	3	−1.27 ± 0.57^e^	5	1.24 ± 0.13^c^	3
F3	1.02 ± 0.54^c^	4	−1.33 ± 0.68^e^	6	0.45 ± 0.27^d^	4
F1S	−0.89 ± 0.12^e^	5	1.58 ± 0.13^ab^	2	−0.29 ± 0.12^e^	5
F2S	4.08 ± 0.22^a^	1	2.76 ± 0.21^a^	1	3.76 ± 0.21^a^	1
F3S	2.47 ± 0.09^b^	2	0.05 ± 0.06^cd^	4	1.88 ± 0.08^b^	2

*Values are presented as mean ± standard error from 10 replicates each treatment. Different letters indicate significant differences among treatments (Tukey’s test, P ≤ 0.05).*

## Discussion

Photosynthesis is a unique physiological process in plants, which affects basal metabolism. It is also an important environmental factor affecting plant growth, development, reproduction, and distribution (Shen et al., 2019; Xiao et al., 2019). In this study, fertilization significantly increased the photosynthetic performance of *P. massoniana* seedlings (Table 2), although both lower and higher fertilization rates have been reported to reduce the photosynthetic performance of plants (Blevins et al., 2006; Boyce et al., 2006). Among the three fertilization treatments applied, the highest values of *P*_*n*_, *T*_*r*_, *G*_*s*_, and WUE were recorded under F2S (Figure 1). Under the same water conditions, SAP application can improve water uptake and retention and provide a better soil moisture environment for seedling growth and development, which improved the *P*_*n*_, *T*_*r*_, *G*_*s*_, and WUE (Islam et al., 2011; Yang et al., 2017). In addition, the mixture compound fertilizer and SAP application effectively alleviated the adverse effects of mild drought on plant photosynthesis (Zhao et al., 2013; Yang et al., 2019; Li et al., 2020), indicating that F2S improved the physiological characteristics of the seedlings compared with those with the other treatments. Under the same treatment, the *P*_*n*_, *T*_*r*_, and *G*_*s*_ in July 2018 were higher than the values in July 2019 (Table 2). A possible explanation for these results is the 1-year-old needles of *P. massoniana* in vigorous growth and development period, resulting in relatively high values of *P*_*n*_, *T*_*r*_, and *G*_*s*_ (Prado and Damascos, 2001; Whitehead et al., 2011; Chen Y. S. et al., 2020). Conversely, the WUE in July 2019 was higher than that in July 2018 (Figure 1) and was negatively correlated with *T*_*r*_ and *G*_*s*_ (Figure 6). The WUE increased as *T*_*r*_ and *G*_*s*_ decreased.

Plant growth is adversely affected by many abiotic factors (Luo et al., 2016), and alleviating such factors can effectively promote vegetative growth. Many studies have shown that improving soil fertility can promote plant growth (Ge et al., 2019; Qiao et al., 2019; Ji et al., 2020). Fertilization significantly affects major plant growth indicators, including ground diameter, plant height, and biomass, among others (Khasa et al., 2001; Vaario et al., 2009). In this study, fertilization significantly increased the RCD growth and HG of the seedlings (Figure 2), in addition to the root, stem, and leaf biomass (Figure 4). Studies have shown that fertilization promotes seedling growth (Li et al., 2016; Yang and Yang, 2020); however, excessive fertilization may not result in significant growth (Óskarsson et al., 2006; Zeng et al., 2013) and could even be detrimental (Uddin et al., 2012). In this study, the RCD growth and HG were the largest under F2S. Meanwhile, the fertilization rate under F3 and F3S was considerably high for seedling growth, and most of the fertilizer may have been lost to the environment (Figures 2, 4). The TN, TP, and TK content in the soil of F3, F2S, and F3S were higher than those of all other treatments (Figure 4). S = Super absorbent polymer can retain a large quantity of water and nutrients when incorporated with the soil, and slowly release stored water and nutrients to improve the growth of plants under limited water and nutrients supply (Islam et al., 2011; Zhang et al., 2013). When growth is limited by nutrient availability, plants produce excess roots to adapt to such nutrient-scarce environments, and the root biomass increases as a result (Tanis et al., 2015). This is consistent with conditions of CK in our study, under which biomass was in the order of root > leaf > stem (Figure 4). However, fertilization decreased the root biomass but increased the stem and leaf biomass relative to the total biomass (Deng et al., 2019). Typically, root biomass decreases in nutrient-rich soils, and fertilization may reduce fine root biomass (Jia et al., 2010); however, our results are consistent with previous reports that the root biomass increased significantly under fertilization (Bolte et al., 2004; Tanis et al., 2015). Under the mixture compound fertilizer and SAP application, the aboveground biomass was significantly higher than the belowground biomass (Table 2). Previous studies have shown that under the same water and fertilizer conditions, SAP application increased the soil water conservation capacity, improved the aboveground plant indices, and reduced the root biomass (Yang et al., 2011; Zhang et al., 2018).

Plants themselves produce structural effects; that is, different tissues exhibit unique functions, growth, and life cycle strategies, resulting in differential nutrient absorption in plant tissues (Deng et al., 2019). Under different fertilization treatments, the distribution of N, P, and K content in the roots, stems, and leaves of the seedlings was variable. The root system is the main tissue of plants for directly absorbing soil nutrients for growth (An et al., 2006). In this study, fertilization significantly increased the N, P, and K content in the roots, stems, and leaves of the seedlings (Figure 5). However, some studies have shown that increased soil nutrient levels often reduce plant nutrient absorption efficiency (Vergutz et al., 2012; Yuan and Chen, 2015). Compared with the single compound fertilizer application, the mixture compound fertilizer and SAP application increased the NUE, PUE, and KUE (Table 5). As a soil conditioner, SAP can improve the soil water-holding capacity and soil aggregation, effectively protect soil nutrients (Busscher et al., 2009), and promote nutrient absorption and utilization by plants (Liu et al., 2013). We observed that the NUE first increased and then decreased with an increase in the fertilizer amount, with the highest value recorded under F2S (Table 5). Therefore, the mixture compound fertilizer and SAP application augmented the nutrient use efficiency of *P. massoniana* seedlings. However, conventional *P. massoniana* fertilization in our study area is largely based on the single or combined application of N and phosphate fertilizers (Qiao et al., 2019; Wu et al., 2019; Huang et al., 2019). Therefore, reasonable and effective fertilization practices must be established to improve the NUE. The PUE was significantly lower than NUE and KUE (Table 2), perhaps due to the extremely low absorption efficiency of P in acidic soils (Erro et al., 2011). According to the literature, most *P. massoniana* forests in the red soil regions of southern China are characterized by P and N deficiency (Chen et al., 2011; Mao et al., 2018). Notably, the KUE was much higher than PUE (Table 2). The deficiency of available potassium in natural red soils in southern China (Zeng et al., 2013) likely promoted fertilizer absorption and utilization by these seedlings. In this study, the higher fertilization rate did not promote nutrient uptake, thus decreasing the FUE (Table 2). Therefore, under specific absorption capacity, excess fertilization exceeded plant demand, resulting in low nutrient uptake, which ultimately reduced FUE (Goodman et al., 2013).

In this study, our results showed that TN, TP, AP, TK, and AK in the soil had a significantly positive correlation with the photosynthesis, growth, and nutrient content in the seedlings (Figure 6B). The compound fertilizer application to soils did great in improving the soil N, P, and TK cycling, which in turn influenced the plant nutritional status and growth (Ge et al., 2019). And the N:P ratio in the soils and plants was significantly negatively correlated with the growth and nutrient of the seedlings (Figure 6B). The N:P ratio can be used to diagnose whether the N nutrient and the supply of soil nutrients is limited during growth (Zhang et al., 2013), and indicates changes in plant growth (Deng et al., 2019). Our study concluded that the N:P ratio in the soil was much lower than that in the *P. massoniana* plantation studied by Lei et al. (2017), which indicated that although there were more P elements given to the soil by the environment, its effectiveness was low. In addition, this study showed that the N:P ratio in the leaves, stems, and roots was slightly less than 14 in all groups, indicating that the growth of *P. massoniana* was limited by the N element to some extent (Ge et al., 2019). Moreover, the results of the comprehensive analysis of the different treatments showed that the comprehensive score for F2S was the highest (Table 6). Therefore, moderate mixture compound fertilizer and SAP application may promote *P. massoniana* growth. In addition to its effects on the plant growth and yield, fertilization affects many ecological aspects of forest plantations. Over 90% of the environmental impact is caused by fertilizer decomposition, nutrient leaching, and runoff during fertilization (Gorecki, 2003). Based on the results of this study, reduced fertilization may promote plant growth and fertilizer utilization. Therefore, to balance plant growth and environmental sustainability, the fertilization amount and method should be designed to promote plant nutrient absorption and growth.

## Conclusion

Under the field experimental conditions, compared with CK, fertilization significantly improved the *P*_*n*_, *G*_*s*_, *T*_*r*,_ WUE, ground diameter, plant height, biomass, soil chemical properties, and nutrient content of the *P. massoniana* seedlings. Compared with other fertilization, the mixed application compound fertilizer and SAP achieved favorable results, having the highest RCD and HG growth. The TN, TP, AP, TK, and AK in the soil had a significantly positive correlation with the photosynthesis, growth, and nutrient content in the seedlings under different fertilization treatments. The comprehensive analysis of the growth characteristics and FUE of the seedlings showed that the mixture compound fertilizer and SAP application (F2S) may serve as a highly effective fertilization method for *P. massoniana* growing in the severely eroded and degraded red soils of southern China. Compound fertilizer, especially when combined with SAP, was more efficient than single compound fertilizer application for *P. massoniana* forests with severely eroded and degraded red soils region.

## Data Availability Statement

The raw data supporting the conclusions of this article will be made available by the authors, without undue reservation.

## Author Contributions

LM: conceptualization, data curation, methodology, investigation, formal analysis, and writing. RZ: visualization, investigation, software, writing, reviewing, and editing. JZ: conceptualization, methodology, writing, reviewing, and editing. SC: conceptualization, methodology, writing, reviewing, and editing. LJ: analyzed the data. XZ: writing, reviewing, and editing. All authors contributed to the article and approved the submitted version.

## Conflict of Interest

The authors declare that the research was conducted in the absence of any commercial or financial relationships that could be construed as a potential conflict of interest.

## Publisher’s Note

All claims expressed in this article are solely those of the authors and do not necessarily represent those of their affiliated organizations, or those of the publisher, the editors and the reviewers. Any product that may be evaluated in this article, or claim that may be made by its manufacturer, is not guaranteed or endorsed by the publisher.
